# Comparison of the Glenoid Index by Computed Tomography With Magnetic Resonance Imaging

**DOI:** 10.7759/cureus.51914

**Published:** 2024-01-08

**Authors:** Nida A Ahmed, Kailash Narendran, Nishath A Ahmed, Prashanth A, B Holebasu, Mihit Kalawatia, Kunal Dudeja, Parijat Kamble, Roshan Prasad, Gaurav Mittal, Ravi Sangoi

**Affiliations:** 1 Trauma and Orthopaedics, Barnsley Hospital NHS foundation Trust, Barnsley, GBR; 2 Internal Medicine, Manipal Hospital, Bangalore, IND; 3 Pediatrics, Dr. B.R. Ambedkar Medical College and Research Institute, Bangalore, IND; 4 Physiology, Mahatma Gandhi Institute of Medical Sciences, Wardha, IND; 5 Radiodiagnosis, Gadag Institute of Medical Sciences, Gadag, IND; 6 Medicine, Rajarshee Chatrapati Shahu Maharaj, Kolhapur, IND; 7 Physiology, Maharjah's Institute of Medical Sciences, Nellimarla, IND; 8 General Medicine, Terna Medical College, Mumbai, IND; 9 Medicine and Surgery, Jawaharlal Nehru Medical College, Datta Meghe Institute of Higher Education and Research, Wardha, IND; 10 Research and Development, Rotract Club Of Indian Medicos, Mumbai, IND; 11 Research, Students Network Organization, Mumbai, IND; 12 Internal Medicine, Mahatma Gandhi Institute of Medical Sciences, Wardha, IND; 13 Internal Medicine, Punyashlok Ahilyadevi Holkar Government Medical College and General Hospital, Baramati, IND

**Keywords:** radiography, ct, mri, glenoid bone loss, glenoid index

## Abstract

Introduction

Anterior shoulder instability results in labral and osseous glenoid injuries. With a large osseous defect, there is a risk of recurrent dislocation of the joint, and therefore the patient has to undergo surgical correction. An MRI evaluation of the patient helps to assess the soft tissue injury. Currently, the volumetric three-dimensional (3D) reconstructed CT image is the standard for measuring glenoid bone loss and the glenoid index. However, it has the disadvantage of exposing the patient to radiation and additional expenses. This study aims to compare the values of the glenoid index using MRI and CT.

Methodology

The present study was a two-year cross-sectional study of patients with shoulder pain, trauma, and dislocation in a tertiary hospital in Karnataka. The sagittal proton density (PD) section of the glenoid and enface 3D reconstructed images of the scapula were used to calculate glenoid bone loss and the glenoid index. The baseline data were analyzed using descriptive statistics, and the Chi-square test was used to test the association of various complications with selected variables of interest.

Results

The glenoid index calculated in the current study using 3D volumetric CT images and MR sagittal PD images was 0.95±0.01 and 0.95±0.01, respectively. The CT and MRI glenoid bone loss was 5.41±0.65% and 5.38±0.65%, respectively. When compared, the glenoid index and bone loss calculated by MRI and CT revealed a high correlation and significance with a p-value of <0.001.

Conclusions

The study concluded that MRI is a reliable method for glenoid measurement. The sagittal PD sequence combined with an enface glenoid makes it possible to identify osseous defects linked to glenohumeral joint damage and dislocation. The values derived from 3D CT are identical to the glenoid index and bone loss determined using the sagittal PD sequence in MRI.

## Introduction

The glenohumeral joint is dynamic due to its extensive range of motion. Because of its anatomical configuration, this joint is prone to dislocation, and numerous elements are required to keep it stable [1]. Four different articulations, glenohumeral, acromioclavicular, sternoclavicular, and scapulothoracic, work favorably together to provide joint flexibility. Glenoid dysplasia, a prominent developmental defect characterized by flatness and slope, involves osseous hypoplasia of the posteroinferior glenoid border and is linked with hypertrophy of the glenoid labrum and surrounding cartilage [2].

An auxiliary ossicle, the os acromiale, can form between the distal aspect of the clavicle and the acromion process. This variant affects between 1% and 15% of the population and can induce impingement and clinical symptoms when present, or it might be asymptomatic. An os acromiale may be clinically important if there are other abnormalities on MRI, such as bone marrow edema and rotator cuff tendinopathy [3]. The inferior glenohumeral ligament (IGHL) complex is made up of the anterior and posterior ligaments. Because of the thickness of the anterior ligament, an axillary recess, or pouch, arises between them. The glenoid labrum is the root cause of IGHL. The antero-inferior labrum forms the anterior band, while the postero-inferior labrum forms the posterior band. These two broad ligaments have their insertions on the anterior and posterior surfaces of the humeral surgical neck, respectively [4].

Huvrov et al. mentioned many components that aid in shoulder mobility, including the sternoclavicular and costoclavicular joints, which connect the upper limb to the axial skeleton and improve joint alignment. The extensive soft-tissue contact of the scapulothoracic joint facilitates movement between the thorax and ventral scapula. The glenoid fossa and the humeral head's limited surface area allow movement in the glenohumeral joint [5]. Several factors influence shoulder joint stability, including misalignment of the glenoid and humeral head articular surfaces, ligament tension, scapula-thoracic and rotator cuff muscles, and capsule volume [6].

For a control system to produce a reflex and keep the joint stable, it needs a sensor system and a feedback mechanism. The glenoid labrum is the best location for sensors, but as of right now, there is no evidence that labrum sensors exist [7]. In addition to a number of other non-modifiable risk factors, acute traumatic shoulder injuries are the main cause of glenohumeral joint instability. Age-related declines in the risk of injury are most common in the second and third decades of life. In general, men are more likely to experience glenohumeral instability than women. Athletes who participate in contact sports, such as football players and wrestlers, are more vulnerable to periods of glenohumeral joint instability [8].

An MRI is used to assess soft tissue damage following a dislocation of the shoulder. The most accurate way to detect soft tissue injury is with this approach, which may also help to identify associated bone damage. Bone-related imaging can be distinguished using T1-weighted imaging as well as nonfat-saturated and fat-saturated combinations of T2-weighted and proton-density sequences in the axial, coronal, and sagittal oblique planes. When joint effusion is present in an acute situation, magnetic resonance arthrography is not particularly helpful, three-dimensional (3D)-reconstructed CT face images of both glenoid bones are required. These pictures can be used to calculate the maximum height and maximum width of the glenoid [9].

By using CT and humeral head subtraction, 3D reconstruction can determine the extent of bone loss. Sagittal glenoid scans from MRI, which are comparable to 3D CT, have been proven in recent studies to be useful in assessing bone loss. The breadth of the patient's glenoid is compared to the diameter of a perfect circle projected onto the inferior glenoid. To calculate the percentage of bone loss, divide the difference between these two measurements by the diameter of the perfect circle. An alternate method for determining the optimal glenoid width is to measure the glenoid's height on a sagittal MRI scan and calculate one-third of glenoid height plus 15 mm for men or one-third of glenoid height plus 13 mm for women [10]. Early surgical intervention should be considered for young, competitive athletes. Bone transplantation is considered if the glenoid lesions cover more than 20%-25% of the articular surface [11].

For glenoid width assessment and choosing the best surgical intervention, the glenoid index is a helpful metric. The purpose of this study was to compute the glenoid index using both CT and MRI imaging methods so that the outcomes of each could be thoroughly compared.

## Materials and methods

Study design and population selection

This study adopted a cross-sectional and observational design. The reporting and article preparation for the cross-sectional aspects of the study adhered to the Strengthening the Reporting of Observational Studies in Epidemiology (STROBE) recommendations. The research was conducted at the department of radiology, A J Institute of Medical Sciences, Mangalore, a tertiary-care hospital in Karnataka, India. The study population comprised 71 individuals aged 10 years or older who presented with shoulder symptoms such as discomfort, dislocation, trauma, and restricted shoulder movements. All participants provided written informed consent. Exclusion criteria encompassed individuals with heart pacemakers, intracranial aneurysmal clips, implanted hearing aids, or metallic bodies in the eye, as well as those with claustrophobia. The commencement of the research occurred only after obtaining the requisite approval from the institutional ethics committee of the AJ Institute of Medical Sciences and Research Centre, Mangalore. This robust ethical oversight ensures the protection of participants' rights and welfare throughout the study.

Data sources and measurement of variables

The below formula determines the required total sample size.

$N = \left[\frac{Z_\alpha + Z_\beta}{C}\right]^2 + 3$

Zα was 1.9600, and the normal standard deviation for Zα, which had been set to 0.05, is the threshold probability for rejecting the null hypothesis. Zβ was 0.8416, representing the probability of failing to reject the null hypothesis under the alternate hypothesis.

The value for C was determined using the below formula.

$C = \left(\frac{1+r}{1-r}\right){0.5}$, yielding 0.4236

r represented the expected correlation, which was $0.4 \times 28$

Based on the above formula, N was determined to be 65. However, our study included 71 subjects (considering non-responses).

Magnetic resonance imaging technique

Multiparametric MRI (mpMRI) of the shoulder was conducted on a 1.5-T unit (Magnetom Avento, Siemens Medical Solutions, Malvern, PA) using a phased array shoulder coil. The imaging protocol included proton density (PD)-weighted and T2-weighted shoulder sequences acquired in appropriate planes, both with and without fat saturation.

Conventional MRI images were obtained through the following sequences: 1. axial PD oblique sequence; 2. axial proton density fat-saturated (PDFS) oblique sequence; 3. PDFS-weighted coronal oblique images; 4. sagittal PD oblique FS sequence; 5. sagittal PD oblique sequence of the opposite shoulder

Additionally, axial CT sections of the bilateral shoulder were obtained, and a 3D volumetric reconstruction of the scapula with glenoid enface was performed. This comprehensive imaging approach provides a detailed assessment of the shoulder anatomy and pathology, allowing for a thorough evaluation of the structures and tissues involved.

Formulas used to calculate the glenoid index

For CT data, $GI = \frac{W'}{W2}$, where W’ is the width of the glenoid before the injury and W2 is the width of the injured glenoid.

For MRI data, $GI = \frac{W'm}{W2m}$*,* where W’m is the width of the glenoid before the injury and W2 is the width of the injured glenoid.

The formula used to calculate the percentage of glenoid bone loss (PGBL) is presented below.

$PGBL = \left(\frac{\text{Defect of width}}{\text{Diameter of the inferior circle}}\right) \times 100$

Glenoid bone loss

To determine the percentage of glenoid bone loss, the best-fit circle method was utilized. In this method, a circle is drawn along the inferior part of the glenoid, and a line is drawn through the center of the glenoid. The drawn line represents the maximum width (D), touching the maximum width of the circle. The measurement of osseous loss (d) is then obtained as the line between the circle's anterior margin and the anterior margin of the inferior glenoid. This approach provides a quantitative assessment of bone loss in the glenoid, which is crucial for evaluating and planning appropriate interventions.

$PGBL = \left(\frac{d}{D}\right) \times 100$

Statistical data analysis

The collected data were analyzed using statistical software, including IBM SPSS version 26.0 (IBM Corp., Armonk, NY). Additionally, Python 3.10.0 (Python Software Foundation, Wilmington, DE) and Jupyter Notebook 4.6 (Project Jupyter, Jupyter Team, https://jupyter.org.) were employed for data analysis. Graphs and tables were generated using a combination of Microsoft Excel (Microsoft Corp., Redmond, WA), Tableau 2021.4.3 (Tableau Software, LLC, Seattle, WA), and Python 3.10.0. This integrated approach, utilizing various tools, allows for a comprehensive and multifaceted analysis of the data, ensuring accuracy and efficiency in presenting the results.

## Results

A total of seventy-one patients who had presented with shoulder pain, dislocation, and a history of trauma underwent MR imaging of the shoulder joint. A sagittal PD sequence of the opposite shoulder was acquired for each patient. Following a 3D volumetric reconstruction of the scapula, the bilateral shoulder joint's CT sections came next. Using CT and MRI, it was possible to determine the dimensions of the injured and uninjured glenoid. These parameters were used to calculate the glenoid index and glenoid bone loss.

The present study showed that the mean age of female patients was 48.5 years, while the mean age of male patients was 38.54 years.

Table 1 shows the frequency distribution of age, gender, and history.

**Table 1:** Frequency distribution of patients in the studied population as per age, gender, and history

Variables	No. of patients (N=71)	Percentage (%)
Age (in years)		
<20	4	5.6
20-30	18	25.4
>30	49	69
Gender		
Female	23	32.4
Male	48	67.6
History		
Trauma	18	25.4
Dislocation	18	25.4
Joint pain	21	29.6
Restriction of movements	14	19.7

The age-frequency distribution of the studied population reveales that 49 patients were over the age of 30, constituting 69% of the total. The age range varies from the youngest patient at 11 years old to the oldest patient at 75 years old.

The gender frequency distribution in the studied population indicates a predominance of males, comprising nearly 68% of the total patient cohort.

The frequency distribution based on the history among patients in the studied population highlights joint pain as the predominant complaint, making up 29.6%. Furthermore, trauma and dislocation each account for 25.4% of the presentations.

Table 2 displays the measurements of the uninjured glenoid obtained through CT, with heights and widths of 2.70±0.09 cm and 1.98±0.06 cm, respectively.

**Table 2:** The comparison of CT clinical variables in males and females

CT variables	Gender		Total
	Female	Male	
Height of the uninjured glenoid	2.72±0.16	2.69±0.12	2.70±0.09
Width of the uninjured glenoid	1.99±0.10	1.98±0.07	1.98±0.06
Height of the affected glenoid	2.74±0.16	2.66±0.11	2.68±0.09
Width of the affected glenoid	1.95±0.10	1.87±0.07	1.90±0.06
Pre-injured width of the affected glenoid	2.03±0.10	1.99±0.08	2.00±0.06
CT index	0.96±0.01	0.94±0.01	0.95±0.01

The corresponding measurements from the MRI for the uninjured glenoid were also 2.70±0.09 cm and 1.98±0.06 cm. For the injured glenoid, CT measurements indicated heights and widths of 2.68±0.09 cm and 1.90±0.06 cm, while MRI measurements showed heights and widths of 2.68±0.09 cm and 1.89±0.06 cm, respectively.

Table 3 presents a comparison of clinical variables from MRI, including the height and width of the uninjured glenoid, the height and width of the affected glenoid, the pre-injured width of the affected glenoid, and the CT index.

**Table 3:** The comparison of MRI clinical variables in males and females

MRI variables	Gender		Total
	Female	Male	
Height of the uninjured glenoid	2.71±0.16	2.69±0.12	2.70±0.09
Width of the uninjured glenoid	1.98±0.09	1.98±0.7	1.98±0.06
Height of the affected glenoid	2.74±0.16	2.66±0.11	2.68±0.09
Width of the affected glenoid	1.92±0.09	1.88±0.07	1.89±0.06
Pre-injured width of the affected glenoid	2.01±0.10	1.99±0.07	1.99±0.06
MRI index	0.96±0.01	0.94±0.01	0.95±0.01

The data have been tabulated separately for male and female subjects, as well as in total, providing mean and standard deviation values.

Table 4 displays the CT/MRI bone loss, separately presented for males and females as well as the overall population; mean and standard deviation values are provided as well.

**Table 4:** The CT/MRI bone loss

Variables	Gender		Total
	Female	Male	
CT bone loss	4.13±0.88	6.02±0.86	5.41.±0.65
MRI bone loss	4.23±0.85	5.93±0.84	5.38.±0.65

Table 5 presents the correlation between CT and MRI, accompanied by the calculated p-values indicating significance.

**Table 5:** The correlation of CT vs. MRI

Pair	r-value	p-value
Height of the uninjured glenoid MRI vs CT	0.998	<0.001
Width of the uninjured glenoid MRI vs CT	0.998	<0.001
Height of the affected glenoid MRI vs CT	0.999	<0.001
Width of the affected glenoid MRI vs CT	0.996	<0.001
Pre-injured width of the affected glenoid MRI vs CT	0.995	<0.001
MRI index vs. CT index	0.971	<0.001

The dimensions of the glenoid, as measured by both CT and MRI, exhibit a strong correlation. The p-value <0.001 underscores the high significance and reproducibility of the data.

## Discussion

The study looked at bone loss and the glenoid index and compared the results with CT imaging to see how effectively MRI can quantify the bony defect of the glenoid. An MRI has been the preferred modality to assess soft tissue injuries in the shoulder joint, whereas CT has been considered a gold standard for detecting and calculating osseous defects. The number of osseous defects determines the surgical management of the patient. The Barstow-Latarjet procedure has been used to correct the osseous defect when the bone loss is more than 25% or the glenoid index is less than 0.75. The patient's exposure to radiation during CT imaging to determine the glenoid loss lengthened his or her stay in the radiology department and increased the cost.

The present study enrolled 71 patients who had complaints of shoulder pain, dislocation, and trauma. Of these patients, 23 were female and 48 were male. The oldest patient was 75 years old, and the youngest was 11 years old. Most patients (29.6%) had presented with joint pain, and the mean age of presentation was 54.3 years. 19.7% of patients presented with restriction of movement, and their mean age was 54.8 years. This outcome is consistent with those of a study by van der Windt et al., who discovered a higher prevalence in the fifth to seventh decade. Their study included 349 patients, and 62% of those subjects were over 45 years of age. They concluded joint pain without a history of trauma and dislocation was common in individuals over 40 years old [12].

A set of 36 patients (50.4%) presented following trauma and dislocation. The mean ages at presentation were 32.7 years and 26.1 years for trauma and dislocation, respectively. Male patients amounted to 66.6% (12) of those who had trauma and 94.4% (17) of patients with dislocation. These findings are in line with those of a study by Zacchilli et al., which suggested that males are more likely to sustain glenoid injury and dislocation. In their study, 71.8% of males presented with shoulder complaints, whereas females constituted 28.2% of the population. The mean age of presentation in their study was 35.4 years for acute presentation, and the risk of injury to the glenoid decreased with age [13].

The uninjured glenoid measured 2.70±0.09 cm in height and 1.98±0.06 cm and width, respectively. The injured glenoid's height and width were 2.68±0.09 cm and 1.90±0.06 cm, respectively, according to CT imaging, and they were 2.68±0.09 cm and 1.89±0.06 cm, respectively, according to MRI imaging.

When compared, the dimensions of the glenoid calculated using MRI and CT demonstrated high correlation and significance, with a p-value <0.001. Sugaya et al. [14] demonstrated that the glenoid dimensions observed on sagittal MRI images align with those identified by 3D CT or two-dimensional (2D) CT, confirming a similarity in findings [15]. The mean glenoid heights measured 39.4±3.7 mm from CT and 39.2±3.6 mm from MRI in a study by Stillwater L et al. measuring the dimensions of glenoids by CT and MRI. The mean glenoid width from CT measured 25.0±3.3 mm; from MRI, this width measured 24.7±3.1 mm [16]. The CT-derived glenoid index calculated in the current study was 0.95±0.01, and the MRI-derived glenoid index was 0.95±0.01. The CT-derived glenoid bone loss was 5.41±0.65%, and the MRI-derived glenoid bone loss was 5.38±0.65%.

With a p-value of 0.001, the glenoid index and bone loss values calculated by MRI and CT showed a strong correlation and significance. In the present study, the supraspinatus tendon was most commonly injured, representing 54.9% of the total rotator cuff injuries. The next most commonly injured tendons were the subscapularis and infraspinatus tendons (22.5% each). These results agree with those of Yamaguchi et al. [17], who said that the supraspinatus tendon tears most often because it has few blood vessels and a thin articular surface. Mall et al. [18] conducted an independent systematic review of nine studies and found that the most common mechanism of injury was falling onto an outstretched arm. The supraspinatus was involved in 84% of tears, and the infraspinatus was torn in 39% of shoulders.

The prevalence of rotator cuff injury was highest (59%) among those aged 50-70 years, which aligns with findings reported by Moor et al. [19]. A total of 599 individuals, corresponding to 607 shoulders, satisfied the specified criteria for inclusion, which pertained to rotator cuff injuries. The average age of the 251 women and 348 men was 56.7 years. As individuals get older, the cuffs gradually deteriorate and become unable to resist pressure, with the occurrence of the superior labrum from anterior to posterior (SLAP) damage frequently linked to dislocation and trauma accounting for 57.1% of the total cases. In a study conducted by Snyder et al. [20], 140 cases of SLAP injuries were identified; 98 cases (70%) had presented with dislocation and trauma, whereas 42 cases (30%) had presented with the insidious onset of pain and discomfort while lifting heavy objects. These authors observed that SLAP lesions usually occur due to inferior traction on the shoulder. Excessive tension and twisting of the long head of the biceps tendon also resulted in injury.

While this study has provided valuable insights into the accuracy of MRI in calculating bony defects of the glenoid and has compared MRI with CT, several limitations also exist. This study included a relatively small sample of 71 patients; a larger and more diverse sample would have enhanced the generalizability of the findings. There was also a significant gender imbalance in the sample, which contained 48 males and 23 females. This disparity may affect the generalizability of the results, especially if gender-specific variations in the assessment of glenoid defects exist. The age range of the participants varied widely, from 11 to 75 years old. Age-related variations in anatomy and pathology could have impacted the study’s outcomes, and subgroup analyses based on age may be necessary in the future. Finally, the study was conducted at a single institution; therefore, its findings may not be universally applicable because different healthcare settings, populations, and equipment can influence the results.

## Conclusions

The study concluded that MRI is an accurate tool for calculating the dimensions of the glenoid. There are osseous defects that can be linked to injury and dislocation of the glenohumeral joint in a sagittal PD sequence with an enfaced glenoid. The glenoid index and bone loss calculated using a sagittal PD sequence in MRI were equivalent to the values calculated using 3D CT.

Therefore, MRI can act as a tool for detecting soft tissue injuries and accurately calculating bone loss to guide the appropriate management of the patient. An MRI has the added advantage of the absence of radiation. By using MRI instead of CT imaging to evaluate the osseous defect, the patient will also incur fewer additional costs. However, if a bilateral glenoid injury is present, the glenoid index and population, intervention, control, and outcomes (PICO) method cannot be applied to calculate the amount of bone loss; the best-fit circle method can be used in this situation.
